# Reliability of coracohumeral distance and subcoracoid tendons in subacromial pain syndrome

**DOI:** 10.1038/s41598-023-29601-0

**Published:** 2023-02-10

**Authors:** Claudia Cavaggion, Santiago Navarro-Ledesma, Birgit Juul-Kristensen, Alejandro Luque-Suarez, Lennard Voogt, Filip Struyf

**Affiliations:** 1 Research Group MOVANT, Department of Rehabilitation Sciences and Physiotherapy, Faculty of Medicine and Health Sciences, University of Antwerp, Campus Drie Eiken, Universiteitsplein 1, 2610 Wilrijk, Belgium; 2 Department of Physiotherapy, University of Granada, Faculty of Health Sciences, Campus of Melilla, Querol Street, 5, 52004 Melilla, Spain; 3 Department of Sports Science and Clinical Biomechanics, University of Southern Denmark, Campusvej 55, 5230 Odense M, Denmark; 4 Department of Physiotherapy, Universidad de Malaga, Arquitecto Penalosa S/N, 29002 Malaga, Spain; 5 Instituto de Investigacion Biomedica de Malaga (IBIMA), Dr. Miguel Díaz Recio, 28, 29010 Malaga, Spain; 6 Department of Physical Therapy Studies and Research Centre for Health Care Innovations, Rotterdam University of Applied Sciences, Rochussenstraat 198, 3015EK Rotterdam, The Netherlands; 7 Pain in Motion Research Group (PAIN), Department of Physiotherapy, Human Physiology and Anatomy, Faculty of Physical Education and Physiotherapy, Vrije Universiteit Brussel, 1090 Brussels, Belgium

**Keywords:** Medical research, Outcomes research

## Abstract

This study investigated the intra-rater reliability of a novice ultrasound (US) examiner and the inter-rater reliability of two examiners (novice, expert) in the measures of coracohumeral distance at rest (CHD) and at 60° of elevation without (CHD60) or with weights (CHD60w), tendon thickness of the long head of the biceps (LHB) and subscapularis (SCP). Twenty-one patients with subacromial pain syndrome (SAPS) and 20 asymptomatic participants were included. Intra and inter-rater reliability were tested with intraclass-correlation-coefficient (ICC), differences between raters were analyzed with Bland–Altman plots. Intra-rater reliability for CHD, CHD60 and CHD60w was excellent (ICC = 0.97–0.98) in asymptomatic participants, and good-to-excellent (0.88–0.93) in SAPS, while intra-rater reliability for LHB and SCP was good-to-excellent in asymptomatic participants (0.88–0.97) and in SAPS (0.90–0.92). Inter-rater reliability for CHD, CHD60 and CHD60w was moderate-to-good (0.70–0.90) in asymptomatic participants and good (0.85–0.87) in SAPS, in contrast inter-rater reliability for LHB and SCP was poor in asymptomatic participants (0.10–0.46) and poor-to-moderate (0.49–0.61) in SAPS. Bland–Altman plots revealed systematic and/or proportional bias for tendons’ thickness. A novice showed good-to-excellent intra-rater reliability in all US measures, whereas in comparison to an expert a novice can measure reliably CHD, CHD60 and CHD60w, but not LHB and SCP, where more training is recommended.

## Introduction

Shoulder pain is a common self-reported musculoskeletal complaint with a median prevalence of 16% in general population, which increases in women and high-income countries^1^. It can lead to sick leave and difficulties in daily activities and it has a lifetime prevalence between 7 and 67%^2^. Subacromial pain syndrome (SAPS) is a non-traumatic unilateral shoulder complaint around the acromion elicited during or after arm elevation^3^. On the other hand, shoulder impingement syndrome identifies the cause of shoulder pain in the compression or impingement of subacromial structures under the acromion, as consequence of narrowing of the subacromial space^4^. This mechanism of compression has been questioned as the impingement cannot sufficiently explain the pathology and SAPS is the preferred term, which includes different clinical and/or radiological terms, such as supraspinatus tendinopathy, partial rotator cuff tear, calcific tendinopathy and subacromial bursitis^3^. The subacromial space has been quantified as acromio-humeral distance (AHD), which can be measured through radiographs, magnetic resonance imaging (MRI), computed tomography (CT) scans and ultrasound (US), with the US as recommended measurement method^5^.

An uncommon and underrecognized cause of anterior shoulder pain is subcoracoid impingement^6,7^. Subcoracoid impingement is defined as compression of soft tissues between the coracoid and the lesser tubercle of the humerus due to a reduction of the subcoracoid space, indicated by a decreased coracohumeral distance (CHD)^8^. Soft tissues at risk for impingement in this space include the subscapularis tendon (SCP), tendon of the long head of the biceps (LHB), and the middle glenohumeral ligament^6^. Although it may occur alone, subcoracoid impingement has similar symptoms to subacromial impingement^9^. Interestingly, a moderate correlation was present between AHD and CHD measures in both controls and patients diagnosed with impingement syndrome^10^, where the symptomatic group presented significantly smaller AHD and CHD compared to the control group. Misirlioglu et al. showed that the 35% of patients undergoing arthroscopic subacromial decompression in their study suffered from both subacromial and subcoracoid impingements, and the CHD significantly increased after surgery, together with significant symptoms improvement^9^. Subcoracoid and subacromial spaces were also found altered concurrently in patients with a combination of subscapularis, supraspinatus, and infraspinatus tendon tears^11^. Subcoracoid space, quantified as CHD, might be a contributing factor in SAPS, as Navarro-Ledesma et al. recently found significant differences in CHD at 0° and 60° between both affected and non-affected shoulders in comparison to healthy controls and no relevant differences between groups in the AHD^12^.

The CHD has been measured on MRI^6,8,10,13^ and on US^13–15^, showing good correlation (ICC = 0.78) between these imaging modalities in neutral arm position and moderate correlation (ICC = 0.61) in internal rotation^13^. The biplane fluoroscopy is an emerging tool which allowed an extremely accurate detection of AHD^16,17^ and CHD^18^ during arm movements. However, radiation exposure and high examination costs limit the use of this technique in clinical practice. Ultrasound is instead a non-invasive and safe imaging modality which is more affordable. However, it is highly operator-dependent and therefore experience and training are important factors to be considered during US examination. Intra-rater reliability of CHD was excellent in patients with anterior shoulder pain, when measured by a physiotherapist with advanced training in musculoskeletal imaging^14^, and it was also excellent in patients with rotator cuff tears, when measured by a musculoskeletal radiologist^13^. Likewise, excellent intra and inter-rater reliability were found in asymptomatic population for measuring the LHB thickness in transverse view, where examiners were physical medicine and rehabilitation residents with a non-specified minimal US experience^19^. Similarly, a physiotherapist with advanced US training showed excellent intra-rater reliability in the measures of LHB and SCP thicknesses in patients with unilateral chronic shoulder pain and also in their non-affected side^20^. Therefore, CHD and subcoracoid tendons showed excellent intra-rater reliability when measured by examiners with moderate experience. However, only one intra and inter-rater reliability study included examiners with minimal experience and it concerned only LHB in asymptomatic subjects^19^. Consequently, there is a gap in the literature on these US measures acquired by novice raters and also on comparison with more experienced examiners, in particular in symptomatic population.

Ultrasound can measure structures dynamically in functional arm positions (elevated and with weights). Intra-rater reliability of AHD at 60° of active elevation was excellent when measured by a physiotherapist with advanced US training in SAPS^21^, and it was also excellent at 70° of passive elevation in asymptomatic population when measured by an experienced medical technologist^22^. Inter-rater reliability of AHD at 60° of abduction in asymptomatic subjects was only moderate between two examiners with 2 years of research-related US experience in AHD measurement^23^. When AHD was measured at 60° of abduction in symptomatic subjects by examiners with remarkable difference in US experience, the inter-rater reliability was poor-to-moderate^24,25^. AHD and CHD showed a moderate significant correlation (r = 0.44) in both controls and patients with SAPS, meaning that these measures are likely to change together^10^. Moreover, measuring AHD and CHD at 60° with weights might inform on the changes induced by weights on the subacromial and subcoracoid spaces respectively. Investigating CHD together with AHD at 60° would add value to the current research, as a recent study showed an increase of both measures during shoulder girdle motor control exercises in healthy men^22^. However, intra-rater reliability of a novice US examiner in CHD at 60° with or without weights and further comparison with expert’s measures are currently missing in the literature and should be further investigated before implementation into clinical practice.

Consequently, the goal of this study was to test: (1) the intra-rater reliability of the novice US examiner in the measures of CHD at rest and 60° of elevation, with and without weights, LHB and SCP in patients with SAPS and asymptomatic participants, (2) the inter-rater reliability between novice and experienced examiners in the same US measures.

## Methods

This study was performed following the Guidelines for Reporting Reliability and Agreement Studies (GRRAS)^26^ and it examined the reliability of five US measures (CHD at rest, LHB thickness, SCP thickness, CHD at 60° of elevation with and without weights) in both asymptomatic and symptomatic participants. Firstly, a preparation phase consisted in training and pilot testing and then the reliability phase was conducted in two weeks as cross-sectional study. The Ethical Committee of the Antwerp University Hospital approved the study (ref: B300201837376) and all participants gave written informed consent. All methods were performed in accordance with the Declaration of Helsinki.

### Preparation phase

The relevant literature concerning US measurements of CHD at 0°^13,14^ and 60°^14^, LHB^19,20^ and SCP^20^ was searched, together with the consultation of the technical guidelines^27^ and the standard references values^28^ for musculoskeletal US. Rater A (novice US examiner) followed a 3-days US training on these measures under the mentorship of rater B (US expert) on three asymptomatic participants in Augustus 2019. Rater A was a physiotherapist with 3 years of discontinuous US experience in research on the shoulder, gained during Master studies. However, CHD, LHB and SCP thicknesses were new measures for rater A at the time of the training. Rater B had 9 years of clinical experience as physiotherapist with expertise in US, gained during numerous courses on both general and shoulder-specific musculoskeletal imaging and during PhD studies, which concerned the use of US in rehabilitation in chronic shoulder pain. Rater B already had a high intra-rater reliability at the time of the training, shown in previous studies: for the CHD in anterior shoulder pain at 0° and 60° (ICC = 0.99)^14^, for the LHB in both asymptomatic and symptomatic subjects (ICC > 0.83)^20^, for the SCP in both asymptomatic and symptomatic subjects (ICC > 0.96)^20^. Consequently, the raters developed a measurement protocol based on previous literature, under the expertise of rater B. They discussed different approaches (patient and examiner positions, probe orientation) and compared their US findings (landmarks, reference points of measure on the US image) obtained on the three asymptomatic participants. In February 2020 pilot testing was conducted on other three asymptomatic participants to ensure consistency between raters prior to the start of the reliability phase. After this pilot testing, the reliability phase lasted two weeks and two groups of participants were tested (asymptomatic participants and patients with SAPS).

### Participants

A convenient sample of patients was recruited at three physiotherapy practices in Belgium in February 2020. Symptomatic participants were included if: they were between 18 and 68 years old; they suffered from shoulder pain on one or both sides for minimum 3 months; they tested positive on at least three out of five shoulder provocative tests: Hawkins-Kennedy test, Neer test, Jobe test, painful arc and external rotation resistance test^29^. They were excluded if: they had corticosteroid injections in the past 6 weeks, thoracic or cervical surgery in the past 10 years, previous fractures or surgery on the shoulder examined, recent US imaging detecting full-thickness tears or large calcifications, or if they had competing pathologies, such as shoulder arthritis, neurological disorders and fibromyalgia. Patients were examined only on the affected shoulder and, in case of bilateral pain, on the dominant side, as it is the most affected in case of rotator cuff pathologies^30^. Asymptomatic participants were recruited by emails or words-of-mouth among friends and colleagues. They were excluded if: they had pain in the past 3 months on the shoulder tested, previous shoulder surgery, and if they had tested positive on more than one provocative test. They were examined only on their dominant arm to have one independent measure per US outcome, to be consistent with the selection of the dominant side in the symptomatic group (when possible), and to ensure the same examination time for all subjects.

All eligibility criteria were based on the participant’s self-report with the exception of the provocative tests. All participants filled in the following questionnaires: pain levels on a Numeric Pain Rating Scale (NPRS) at rest, during activity and in the previous week^31^, Shoulder and Pain Disability Index (SPADI)^32^. Both scales are valid and reliable in patients with shoulder pain^31,33^.

### Procedure

Rater A screened the patients and then they completed the questionnaires. Firstly, rater A measured the CHD on the screen three consecutive times. The participant could rest between measurements, if necessary. A sticker was placed on the screen to hide the values and test the intra-rater reliability of a novice US examiner. Also, the other US measures (LHB, SCP, CHD at 60° of elevation with or without weights) were recorded with the same procedures. Afterwards, rater B also scanned and measured three times each of the selected structures (CHD, LHB, CHD at 60° with weights, CHD at 60° with weights), but without the sticker. Raters were blinded to each other’s examinations and findings, but they knew if the participant had SAPS or not. No landmarks were placed on the skin and the probe was repositioned on the shoulder for every measure.

#### Ultrasound protocol

**Figure 1:**
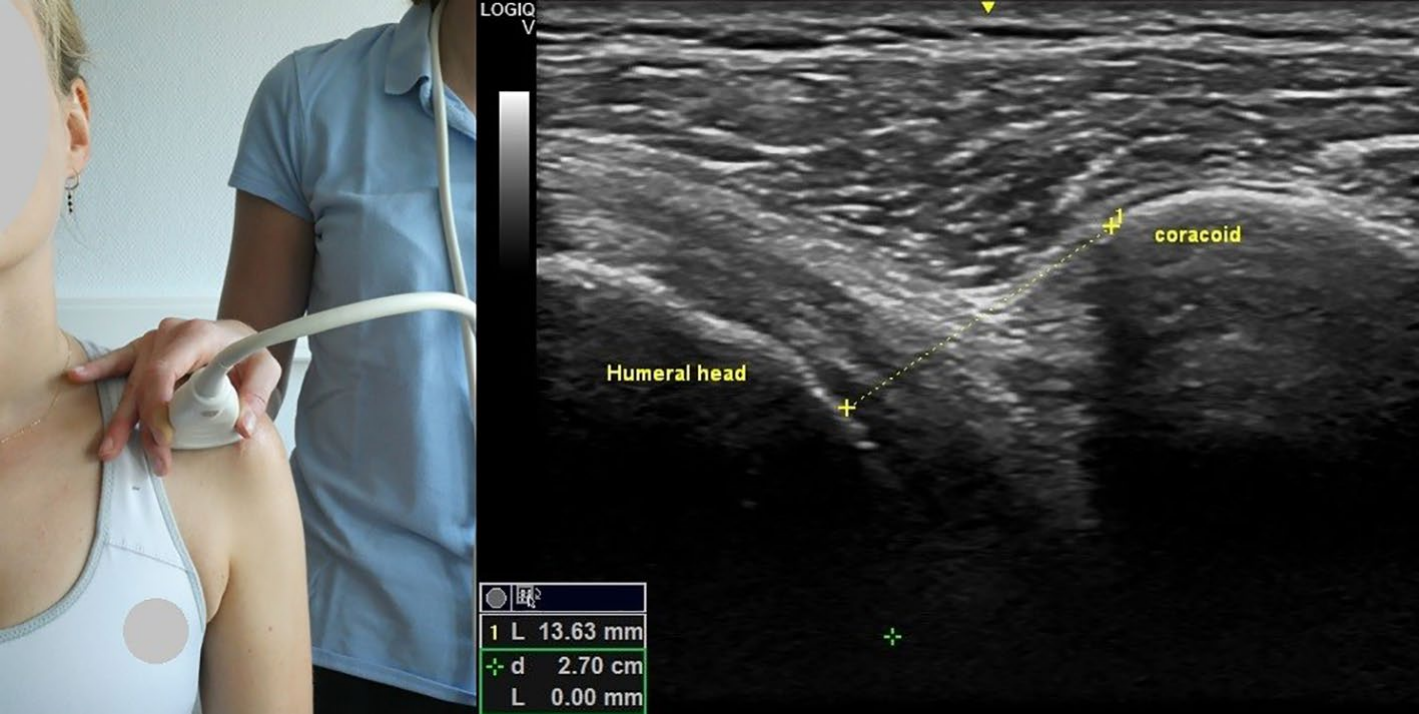
Left: position of the probe during the measurement of the coracohumeral distance at rest; right: measurement of coracohumeral distance at rest. Photo on the left side by Anthe Foubert.

**Figure 2:**
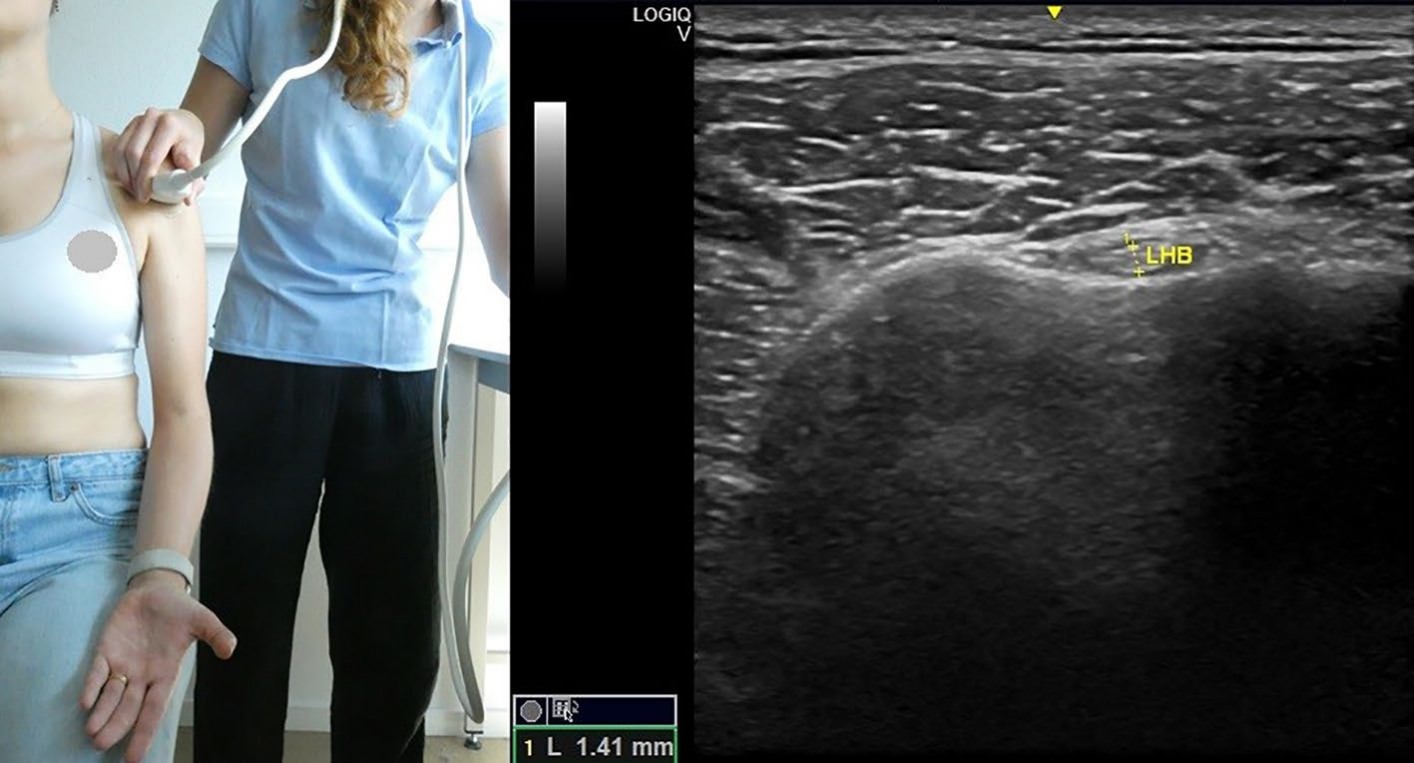
Left: position of the probe and of the subject during the measurement of the long head of the biceps tendon thickness; right: measurement of the long head of the biceps tendon thickness. Photo on the left side by Anthe Foubert.

**Figure 3:**
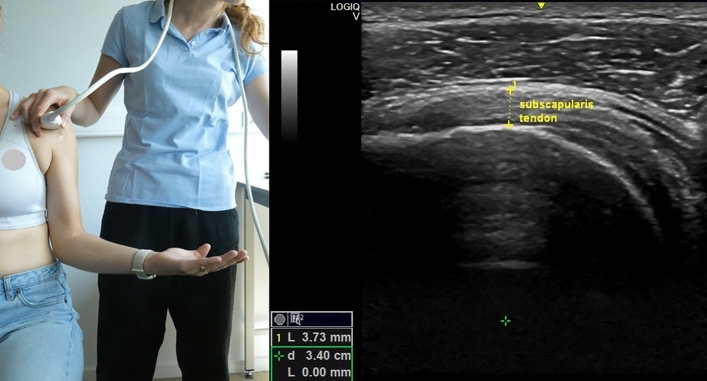
Left: position of the probe and of the subject during the measurement of the subscapularis tendon thickness; right: measurement of the subscapularis tendon thickness. Photo on the left side by Anthe Foubert.

**Figure 4:**
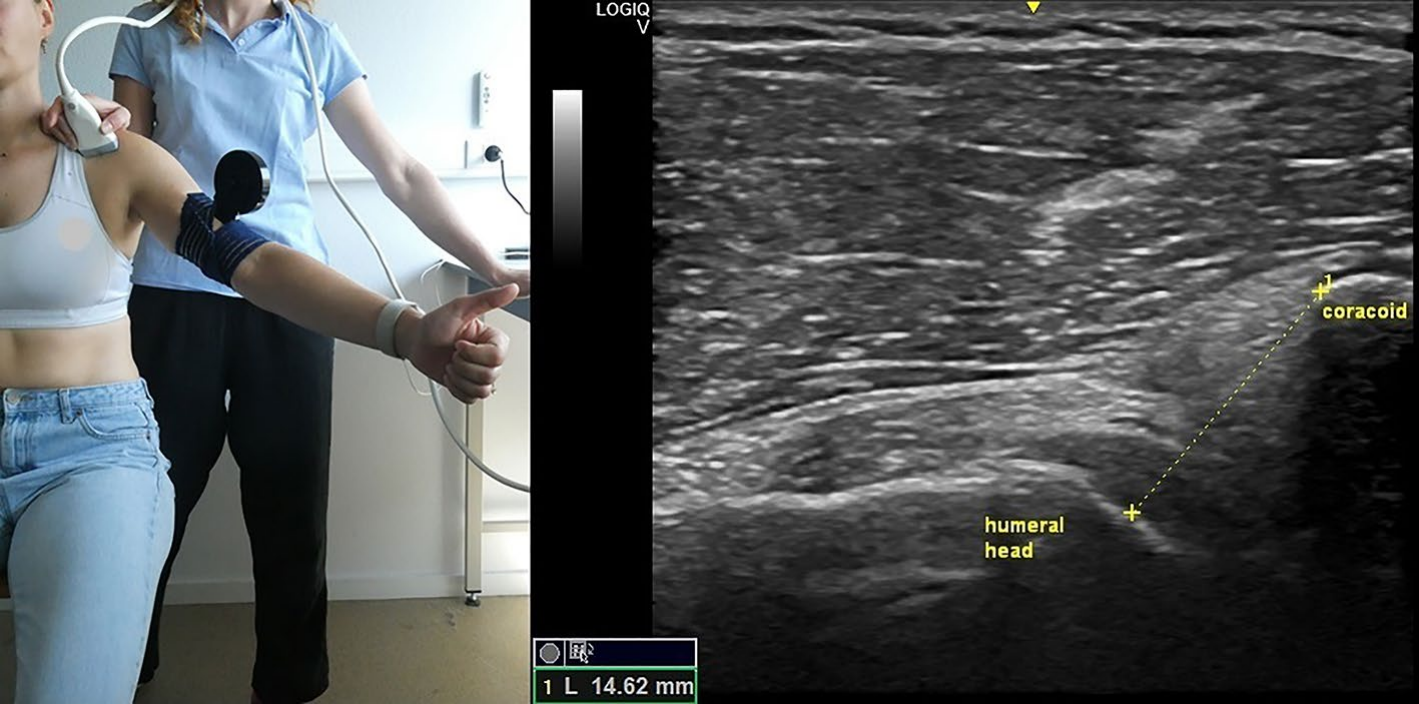
Left: position of the probe and of the subject during the measurement of the coracohumeral distance at 60°; right: measurement of the coracohumeral at 60°. Photo on the left side by Anthe Foubert.

**Figure 5:**
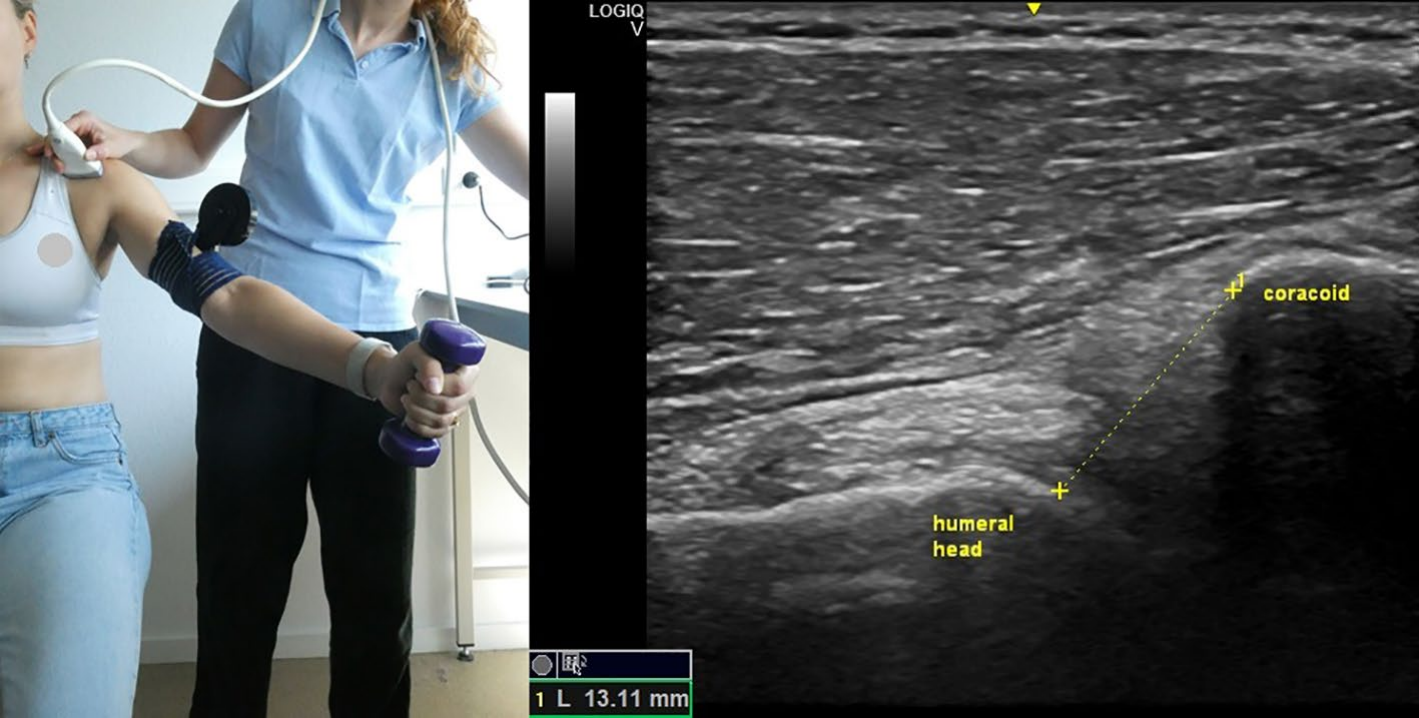
Left: position of the probe and the subject during the measurement of the coracohumeral distance at 60° with weights; right: measurement of the coracohumeral at 60° with weights. Photo on the left side by Anthe Foubert.

The US equipment utilized was GE Logiq-V2 with 4.2–13.0 MHz linear-array transducer (GE Healthcare). A pre-set of different parameters was installed for the study, with depth = 4 cm, frequency = 12 MHz, gain = 51% and with the option “Coded Harmonic Imaging” activated, which enhances field resolution for improving the imaging of small parts. However, the US examiner could change the parameters to obtain a clearer image, if necessary. All US measurements were expressed in millimeters and the participant was always seated in upright position, feet flat on the ground with head straight. Informed consent was obtained by the participants visible in Figs. 1, 2, 3, 4 and 5 for open access publication.

### Coracohumeral distance

For the measure of CHD at rest, the patient was seated with the arm straight resting at the side. The probe was placed on the most anterior aspect of the shoulder and in a transverse position in respect to the humeral axis. The CHD was defined as the shortest distance between the margin of the coracoid process and the humeral head, moving the line across the humeral head until the shortest distance between the two bony landmarks was identified (Fig. 1)^13^.

### Long head of the biceps

The participant’s upper arm was along the body side, with the elbow flexed and the forearm supinated resting on the lap^19^, with the palm up^27^. The probe was placed on the anterior aspect of the shoulder perpendicular to the humerus, and the LHB was visualized between the greater and lesser tuberosities in a transverse view^27^. The thickness of the LHB was measured vertically at the distal end of the rotator cuff^28^, including the thickness of the fascial borders in the measure (Fig. 2).

### Subscapularis tendon

The subject’s initial position and probe’s location and orientation were the same used for the LHB. The participants had to maximally rotate the forearm, fixating the elbow on the iliac crest^27^. The probe was then positioned to identify the SCP centrally on the US image and the thickness of the SCP was measured in this view vertically, excluding the subcoracoid bursa from the measure (Fig. 3).

### Coraco-humeral distance at 60° of elevation

The subject’s initial position and probe’s location and orientation were the same used for the CHD at rest^14^. A liquid damped inclinometer was then fixed on the subject’s upper arm to ensure 60° of shoulder elevation in the scapular plane, and the examiner assisted and corrected the movement if necessary. The subject held the arm position during image capturing, and afterwards the CHD was measured on the screen as the shortest distance between the coracoid process and the humeral head (Fig. 4).

### Coracohumeral distance at 60° of elevation with weights

This measurement was conducted with the same patient’s position and probe’s orientation as in the measure of CHD at 60° of elevation. The participant had to lift a dumbbell according to their body weight: 1 kg for those weighting 70 kg or less, or 2 kg for those weighting more than 70 kg. These measures were based on a previous reliability study on scapular dyskinesis in overhead athletes^34^, using dumbbells of 1.4 kg for those weighing less than 68.1 kg and 2.3 kg for those weighing 68.1 kg or more. Considering that the population in the current study was composed by both asymptomatic and symptomatic subjects (and not overhead athletes), and that the body weight was asked to the participants and not measured, we considered a rounded reference number of 70 kg instead of 68.1 kg. Therefore we used 1 kg for participants having a weight of 70 kg or less and 2 kg for participants having a weight of more than 70 kg. Rater A left the assigned dumbbell in the examination room for rater B. With this measure we aimed to assess the impact of weight on the subcoracoid space (Fig. 5).

### Data analysis

A sample size of 21 participants was calculated according to the following parameters: an expected ICC = 0.90, a minimum ICC = 0.70, α = 0.05, power = 0.80, considering two raters and 10% of drop-out^35^. As differences in reliability may arise in asymptomatic or symptomatic participants, we calculated 21 participants per group for a total of 42.

Data were analyzed in SPSS (Version 28, Windows). All variables were tested for normality using Kolmogorov–Smirnov test (p > 0.05). Differences between SAPS and asymptomatic group were tested with Mann–Whitney U test, because data were not normally distributed, or Chi-squared test. The three trials of each US measure of rater A were reported in SPSS and the intraclass correlation coefficient (ICC) model 3,3 (2-way mixed-effects model—absolute agreement, average measure) was used for intra-rater reliability of rater A. This choice is intended for future applications where the mean of three trials of one rater is considered as assessment basis. For inter-rater reliability, the mean of three trials (or the mean of two trials, in case one measure was missing) for each rater was reported and the ICC model 2,3 (2-way random-effects model—absolute agreement, single measure) was calculated^36^. This model was chosen to generalize the results to other raters with comparable characteristics, but keeping the measurement from a single rater as assessment basis^37^. If an US measurement was lost in the acquisition or processing phases, it was not considered in the analysis of reliability of that specific US outcome. If data obtained from the US measures were not normally distributed, they were transformed into natural logarithm. However, differences between ICCs obtained from raw and transformed data were less than 0.03 and therefore the non-transformed ICCs were analyzed. ICC was interpreted as > 0.90 = excellent, 0.75 to 0.90 = good, 0.50 to 0.75 = moderate, < 0.50 = poor^38^.

Bland–Altman plots with 95% limits of agreement^39^ were conducted to find visually systematic or proportional bias between raters A and B. Differences between raters were tested also with paired t-test, and for data not normally distributed with Wilcoxon sign-rank test. Absence of proportional bias was confirmed also by regression analysis: the regression of the differences on means should have a slope of zero^40^. Finally, the standard error of measurement (SEM) was calculated with the mean square (MS) error term as SEM = √MS_E,_ and the minimal detectable change (MDC) was computed at 95% confidence level as MDC = SEM × 1.96 × √2 for both intra and inter-rater reliability^38^.

## Results

Fifty participants were examined for eligibility but nine patients were excluded for different reasons: no positive testing (n = 3), previous operations or trauma (n = 5), competing pathologies (n = 1). Thus, 41 participants were enrolled: 20 asymptomatic and 21 symptomatic participants. The asymptomatic group had on average 28.0 ± 3.7 years and consisted of ten males and ten females, while the SAPS group had on average 50.2 ± 10.7 years and consisted of three males and 18 females. The SAPS group included significantly older participants and less males (p < 0.05). Pain and dysfunction were significantly higher in the SAPS group (SPADI = 42.9 ± 22.4, NPRS during activity = 4.3 ± 2.5, NPRS in the past week = 5.1 ± 2.2) compared to the asymptomatic group (SPADI = 0.1 ± 0.5, NPRS during activity = 0.0 ± 0.0, NPRS in the past week = 0.1 ± 0.2). The data refers to the same groups of subjects who participated also in another study on other US measures^25^. Means and standard deviations of the three trials for every US parameter for both raters A and B are shown in Table 1, together with the values of the three trials of rater A. The total number of available patients per US outcome is indicated in Table 2 for asymptomatic patients and in Table 3 for the SAPS group. The variation of mean values was similar in intra and inter-rater reliability, with standard deviations of 0.54–3.22 mm and 0.41–3.13 mm respectively.

**Table 1:** US measures (in mm).

	*n*	Rater A	Rater B	Rater A			
		Mean (SD)	Mean (SD)	n	Mean 1 (SD)	Mean 2 (SD)	Mean 3 (SD)
Asymptomatic group							
CHD0	20	14.57 (2.80)	14.21 (2.30)	20	14.59 (3.22)	14.46 (2.69)	14.66 (2.74)
LHB	20	2.67 (0.59)	1.78 (0.24)	19	2.71 (0.70)	2.70 (0.71)	2.49 (0.54)
SCP	19	3.76 (0.89)	4.07 (1.00)	20	3.79 (0.88)	3.92 (0.95)	3.74 (1.00)
CHD60	18	15.42 (2.39)	14.99 (2.32)	19	15.31 (2.47)	15.36 (2.45)	15.17 (2.45)
CHD60w	20	15.33 (2.78)	14.67 (3.13)	20	15.29 (2.87)	15.38 (2.76)	15.32 (2.90)
SAPS group							
CHD0	21	13.49 (2.43)	13.28 (2.71)	20	13.64 (2.67)	13.27 (2.53)	13.37 (2.74)
LHB	21	2.15 (0.59)	1.80 (0.41)	20	2.21 (0.62)	2.12 (0.75)	2.01 (0.54)
SCP	21	3.55 (0.75)	4.18 (0.89)	21	3.58 (0.86)	3.48 (0.69)	3.60 (0.87)
CHD60	21	13.19 (2.68)	12.77 (2.79)	19	12.79 (3.06)	13.20 (2.94)	13.18 (3.11)
CHD60w	21	13.11(2.57)	13.04 (2.68)	21	13.07 (2.73)	13.28 (3.04)	12.99 (2.81)

The 3rd and 4th columns represent the means of three trials of rater A and B respectively. The 6th, 7th and 8th columns represent the first measure of rater A, second measure of rater A, third measure of rater A respectively.

*CHD0* coracohumeral distance at rest, *CHD60* coracohumeral distance at 60°, *CHD60w* coracohumeral distance at 60° with weights, *LHB* long head of biceps tendon, *n* number of participants, *SCP* subscapularis tendon.

**Table 2:** Inter and intra rater reliability in the asymptomatic group.

Asymptomatic group										
	Inter-rater reliability, Raters A and B					Intra-rater reliability, Rater A				
	n	ICC (95%CI)	SEM, mm	MDC, mm	MDC, %	n	ICC (95%CI)	SEM, mm	MDC, mm	MDC, %
CHD0	20	0.70 (0.38; 0.87)	1.42	3.93	27.3	20	0.97 (0.93; 0.99)	0.91	2.51	17.3
LHB	20	0.10 (−0.08; 0.38)	0.38	1.05	47.3	19	0.88 (0.74; 0.95)	0.34	0.95	36.0
SCP	19	0.46 (0.05; 0.75)	0.68	1.90	48.5	20	0.97 (0.93; 0.99)	0.29	0.79	20.8
CHD60	18	0.87 (0.69; 0.95)	0.82	2.26	14.9	19	0.98 (0.96; 0.99)	0.61	1.68	11.0
CHD60w	20	0.90 (0.72; 0.96)	0.85	2.37	15.8	20	0.98 (0.95; 0.99)	0.75	2.08	13.5

*95%CI* 95% confidence interval, *CHD0* coracohumeral distance at rest, *CHD60* coracohumeral distance at 60°, *CHD60w* coracohumeral distance at 60° with weights, *LHB* long head of biceps tendon, *MDC* minimal detectable change at 95% of confidence interval, *MDC%* minimal detectable change in % respect to mean value of raters A and B or to three mean values of rater A, *n* number of participants considered in the statistical analysis, *SCP* subscapularis tendon, *SEM* standard error of measurement.

**Table 3:** Inter and intra rater reliability in the SAPS group.

SAPS group										
	Inter-rater reliability, Raters A and B					Intra-rater reliability, Rater A				
	n	ICC (95%CI)	SEM, mm	MDC, mm	MDC, %	N	ICC (95%CI)	SEM, mm	MDC, mm	MDC, %
CHD0	21	0.85 (0.67; 0.94)	1.01	2.80	20.9	20	0.93 (0.86; 0.97)	1.14	3.15	23.5
LHB	21	0.49 (0.04; 0.77)	0.32	0.89	45.1	20	0.90 (0.78; 0.96)	0.32	0.89	41.8
SCP	21	0.61 (−0.04; 0.86)	0.38	1.05	27.3	21	0.92 (0.83; 0.97)	0.37	1.03	29.1
CHD60	21	0.86 (0.69; 0.94)	1.01	2.79	21.5	19	0.91 (0.81; 0.96)	1.46	4.04	31.0
CHD60w	21	0.87 (0.71; 0.95)	0.97	2.68	20.5	21	0.88 (0.76; 0.95)	1.55	4.30	32.7

*95%CI* 95% confidence interval, *CHD0* coracohumeral distance at rest, *CHD60* coracohumeral distance at 60°, *CHD60W* coracohumeral distance at 60° with weights, *LHB* long head of biceps tendon, *MDC* minimal detectable change at 95% of confidence interval, *MDC%* minimal detectable change in % respect to mean value of raters A and B or to three mean values of rater A, *n* number of participants considered in the statistical analysis, *SCP* subscapularis tendon, *SEM* standard error of measurement.

The intra-rater reliability of rater A was excellent (0.97–0.98) for CHD at rest and at 60° of elevation with and without weights in asymptomatic participants (Table 2), and it was good-to-excellent (0.88–0.93) in SAPS (Table 3). Similarly, intra-rater reliability of rater A was good-to-excellent (0.88–0.97) for LHB and SCP thicknesses in asymptomatic participants, and it was also good-to-excellent (0.90–0.92) in SAPS.

Inter-rater reliability between raters was moderate-to-good (0.70–0.90) for CHD at rest and at 60° of elevation with and without weights in asymptomatic participants (Table 2), and it was good (0.85–0.87) in SAPS (Table 3). Inter-rater reliability for tendons’ thicknesses (LHB, SCP) was poor (0.10–0.46) in asymptomatic participants and poor-to-moderate (0.49–0.61) in SAPS. In general, intra-rater reliability was higher than inter-rater reliability for all US measures, with largest differences in tendons’ thickness. In particular, the inter-rater reliability of tendons’ thickness was lower than measures of CHD at rest or 60° of elevation, with and without weights.

**Figure 6:**
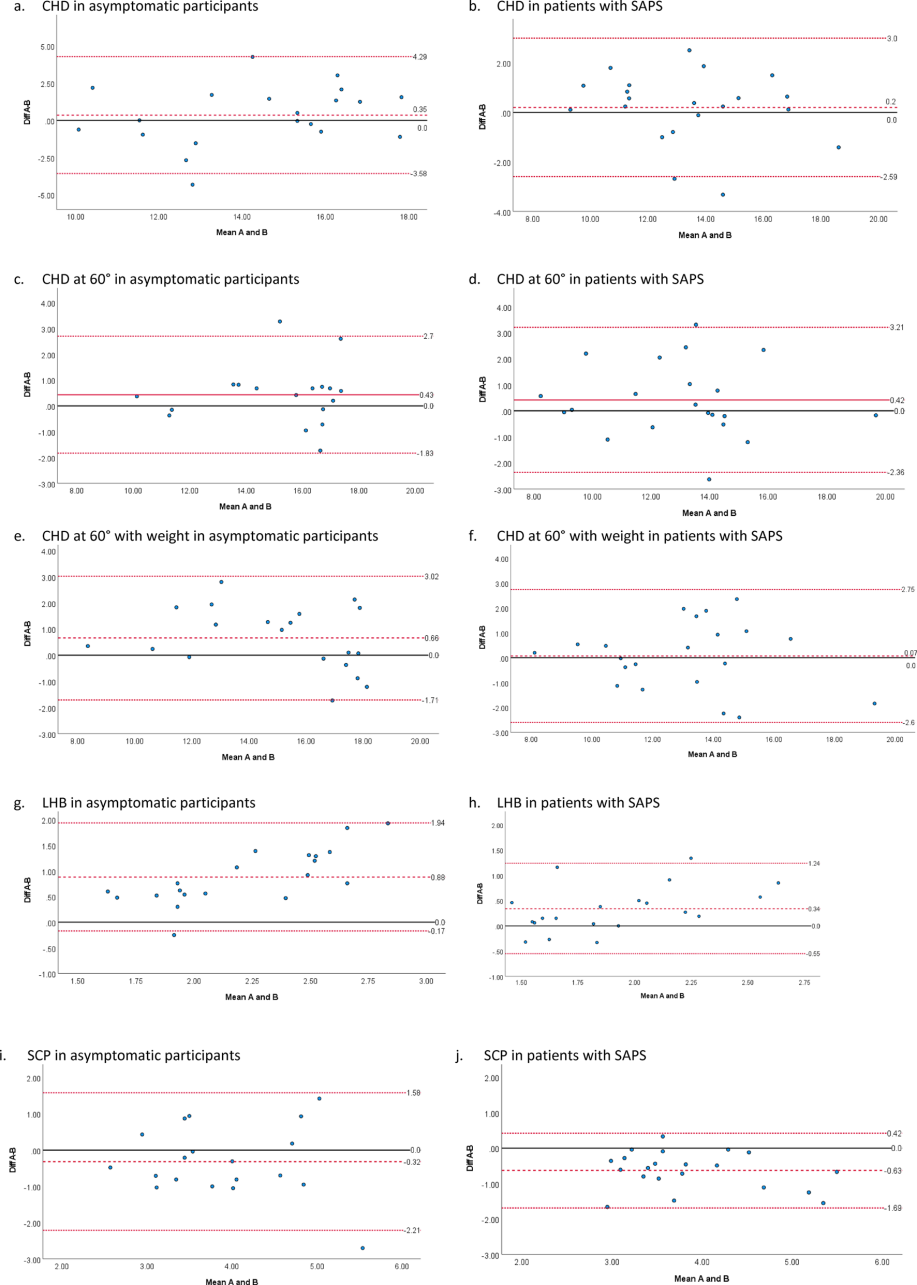
Bland–Altman plots for inter-rater reliability. Color of the lines: dashed red line: mean difference, dotted red line: limits of agreement of the mean difference, black line: perfect agreement (difference between A and B = 0). *CHD* coracohumeral distance, *Diff* difference, *LHB* long head of biceps, *SAPS* subacromial pain syndrome, *SCP* subscapularis tendon.

The SEM and MDC were lower for intra-rater rather than inter-rater values on all US measures in the asymptomatic group, while in the SAPS group they were lower only in SCP and LHB. The MDC in % followed the same trend of SEM and MDC (in mm) in the asymptomatic group, while in the SAPS group it was lower in intra-rater values compared to inter-rater values only in LHB. The Bland–Altman plots showed differences of less than 0.66 mm between raters for CHD at rest and at 60° of elevation with or without weights, and no systematic or proportional bias were present in both asymptomatic and symptomatic participants (Fig. 6a–f). In contrast, rater A evaluated significantly higher LHB than rater B in both asymptomatic (p < 0.001) and symptomatic groups (p = 0.002), and proportional bias was visible in Bland–Altman plots, where rater A evaluated thicker LHB than rater B for participants with thicker LHB (Fig. 6g,h), confirmed by regression analysis (p < 0.042). No significant differences between raters were present in asymptomatic participants for SCP (Fig. 6i), whereas rater A measured significantly lower values (p < 0.001) in SAPS, but no proportional bias was present (Fig. 6j).

## Discussion

This study investigated the intra-rater reliability of a novice US examiner and the inter-rater reliability between two examiners with different US experience in the measures of CHD at rest and at 60° of elevation with and without weights, LHB and SCP thicknesses. Considering all participants and US measures, intra-rater reliability of a novice was good-to-excellent, while inter-rater reliability was poor-to-good.

Intra-rater reliability of the novice examiner showed excellent results in asymptomatic participants in CHD at rest and at 60° of elevation, in line with previous excellent results (ICC = 0.96), where an experienced medical technologist measured the CHD at 70° of passive elevation^22^. Their average CHD was around 5 mm higher compared to our values, probably due to inclusion of only (young) healthy men, who present usually larger CHD than women^6^. However, reliable results were obtained by both an experienced medical technologist and novice (current study) rater in CHD in healthy participants in slightly different positions.

When considering symptomatic participants, intra-rater reliability of CHD at rest was excellent, similarly to Navarro-Ledesma et al., where the examiner was a physiotherapist with advanced US training^14^, or to Oh et al., where the examiner was a musculoskeletal radiologist^13^. However, our current symptomatic group had lateral-superior shoulder pain classified as SAPS, while Navarro-Ledesma et al. investigated anterior shoulder pain and Oh et al. included patients with rotator cuff tears. Consequently, our average CHD in neutral position was higher for both raters (13.3–13.5 mm) than values previously reported (9.9–10.4 mm)^13,14^. At 60° of elevation in the SAPS group, we obtained excellent results in intra-rater reliability, in line with Navarro-Ledesma et al.^14^. Despite various types of shoulder pain, CHD at rest and at 60° of elevation were reliable when measured by single examiners with different US experience.

To our knowledge, this is the first study investigating the inter-rater reliability of CHD at rest and at 60° of elevation without weights, showing moderate-to-good results in an asymptomatic population and good results in a SAPS group. However, three symptomatic participants could not consistently hold the weight assigned, which was downgraded from 2 to 1 kg or from 1 to 0.5 kg. Therefore, in future studies it is suggested to start with a load initially causing slight pain, regardless of the individual body weight.

Reliability of LHB and SCP were investigated in this study as tendons passing through the subcoracoid space. Intra-rater reliability for both LHB and SCP was good-to-excellent in the asymptomatic and symptomatic groups, in line with previous studies^19,20^. Inter-rater reliability of LHB was poor in asymptomatic participants, in contrast with excellent results of Drolet et al.^19^. This may be due to systematic and proportional bias visible in Bland–Altman plots of LHB in both asymptomatic and SAPS groups, with the novice examiner measuring a significantly larger LHB than the expert. The SEM calculated in this study excluded error due to systematic differences between raters, and our MDC of 1.05 mm in asymptomatic participants was similar to 1.0 mm reported by Drolet et al. However, our MDC for LHB was 36% and 47% of the mean values in intra and inter-rater reliability respectively. Therefore, a significant change should be higher than 47% of the LHB initial value when measured by two different examiners and higher than 36% when measured three times by a novice. Consequently, the SEM and MDC of the LHB in the current study should be interpreted with caution. Inter-rater reliability of SCP was poor in asymptomatic participants and moderate in SAPS, where the novice measured significantly lower SCP than the expert. A thorough review of the US images revealed that the novice kept the LHB as reference on the screen, while the expert obtained a full view of the SCP without the need to always visualize simultaneously the LHB. This slight difference in probe positioning could have introduced differences in the SCP measure. Overall, we can note that inter-rater reliability in LHB and SCP thicknesses was generally lower than CHD at rest or at 60° and with or without weights. Tendon borders are indeed more difficult to visualize and anisotropy, depending on the beam angulation^41^, may negatively influence tendon appearance. Moreover, a pre-set of US parameters was installed for the study but the examiner could change them to obtain a clearer image. Although this approach is more similar to daily use of US in clinical practice, it is suggested to use a fixed set of all US parameters in a protocol for reliability studies, in order to avoid the introduction of further differences between raters.

Our study presented some limitations. Firstly, the examiner knew the participant group (SAPS or asymptomatic) during testing and there was no standardized resting period between measurements due to time limits. However, these limitations could have influenced the results, especially in the measures of CHD with weight for patients with SAPS, who sometimes found difficult to maintain the dumbbell at 60° during the US examination. Therefore, it is suggested that a third independent researcher conducts the recruitment and that an interval of at least one minute between measurements is provided in order to improve blinding and reproducibility. Secondly, low ICC and systematic and/or proportional bias in LHB and SCP suggest that a novice does not obtain the same measures of an expert. Therefore, a more rigid US protocol including both asymptomatic and SAPS participants with deeper discussion about measurement procedures is suggested in the pilot phase for LHB and SCP. Lastly, the results of this study concerned a novice and an expert US examiners with different experience and they focused on specific measures in asymptomatic participants and SAPS. Therefore, these results can be generalized only to examiners with similar differences in US experience to this study and in the same type of US measures and population. Including two examiners with similar extensive US knowledge might lead to better results, and adding a third experienced rater might bring more information about inter-rater reliability.

This study had also various strengths. Firstly, no landmarks were placed on the skin aiming to keep independent measures. Secondly, the raters used the same US equipment, so possible differences due to the image processing were excluded. Thirdly, reliability of image-based evaluation includes two aspects that can create variability: capturing and measuring the image^5^, with the first aspect usually giving the poorest reliability. In the current study both raters captured and measured their own images and, although this procedure may have led to poorer results, it gives a complete picture of the US procedure.

The clinical implications of our results should be evaluated not only in light of the ICC but also considering MDC values. In the current study the CHD at rest and at 60° showed good-to-excellent ICCs in the SAPS group when measured by a novice and also when compared to an experienced examiner. However, the MDC was about 24 and 21% of the mean CHD at rest, when measured by a novice or two different raters respectively. Moreover, the MDC was above 31% of the mean CHD intra-rater values and around 21% of the mean CHD inter-rater values, when measured at 60° with or without weights. Despite the promising ICC values, more research is necessary to provide definitive MDC values above measurement error useful in studying significant changes over time and prior to implementation of these measures into clinical practice. Although the current study showed some limitations and high SEM and MDC values, it is a first step to create a definitive measurement protocol of CHD in SAPS.

AHD and CHD showed previously a significant moderate correlation in both controls and patients diagnosed with impingement^10^, suggesting that these variables tend to change together, and their values were considerably higher in the asymptomatic group^10,15^. Navarro-Ledesma et al. recently found a decreased CHD at 0° and 60° in SAPS in both painful and controlateral pain-free shoulder when compared to healthy controls^12^. However, no significant differences in AHD were reported between the three groups. Therefore, the CHD might be a relevant contributing factor in SAPS, as previous research mainly focused on AHD. Nevertheless, SAPS remains a multifactorial disorder where different factors, including biochemical, intrinsic tissue properties and psychological factors, may play a role^12^.

Finally, US gives two-dimensional images of the subcoracoid or subacromial spaces, measured in static positions and influenced by probe and participant positioning. Biplane fluoroscopy is an emerging tool which allows to capture the three-dimensional nature of these spaces, together with accurate in-vivo quantification of shoulder kinematics during dynamic movements^16–18^ and loading. It would be of great interest to investigate both subcoracoid and subacromial spaces during dynamic tasks to reveal possible impingement mechanisms in symptomatic subjects. Nevertheless, fluoroscopy measurements expose the patients to ionizing radiations, on the contrary of US measures. Therefore, it would be valuable to compare measures of subcoracoid and subacromial spaces obtained with US and biplane fluoroscopy, balancing risks and benefits of both analyses.

## Conclusion

Considering all participants, intra-rater reliability of a novice examiner was good-to-excellent for all US measures, while inter-rater reliability between two examiners with different experience (novice and expert) was poor-to-good. In comparison with an expert, a novice can reliably measure the CHD at rest and at 60° of shoulder elevation with and without weights, but not the LHB and SCP tendon thicknesses, where more calibration of procedures is recommended. The SEM and MDC for intra and inter-reliability are provided but more research is necessary before adding these US measures to the clinical evaluation of SAPS.

## Data Availability

The datasets are available from the corresponding author on reasonable request.

## References

[1] Lucas J, van Doorn P, Hegedus E, Lewis J, van der Windt D (2022). A systematic review of the global prevalence and incidence of shoulder pain. BMC Musculoskelet. Disord..

[2] Luime JJ (2004). Prevalence and incidence of shoulder pain in the general population; A systematic review. Scand. J. Rheumatol..

[3] Diercks R (2014). Guideline for diagnosis and treatment of subacromial pain syndrome: A multidisciplinary review by the Dutch Orthopaedic Association. Acta Orthop..

[4] Neer CS (1972). Anterior acromioplasty for the chronic impingement syndrome in the shoulder: A preliminary report. J. Bone Jt. Surg. Am..

[5] McCreesh KM, Crotty JM, Lewis JS (2015). Acromiohumeral distance measurement in rotator cuff tendinopathy: Is there a reliable, clinically applicable method? A systematic review. Br. J. Sports Med..

[6] Giaroli EL, Major NM, Lemley DE, Lee J (2006). Coracohumeral interval imaging in subcoracoid impingement syndrome on MRI. AJR Am. J. Roentgenol..

[7] Lappin M, Gallo A, Krzyzek M, Evans K, Chen YT (2017). Sonographic findings in subcoracoid impingement syndrome: A case report and literature review. PM R.

[8] Leite MJ (2019). Coracohumeral distance and coracoid overlap as predictors of subscapularis and long head of the biceps injuries. J. Shoulder Elbow Surg..

[9] Misirlioglu M (2012). Prevalence of the association of subacromial impingement with subcoracoid impingement and their clinical effects. J. Int. Med. Res..

[10] Hekimoglu B, Aydin H, Kizilgoz V, Tatar IG, Ersan O (2013). Quantitative measurement of humero-acromial, humero-coracoid, and coraco-clavicular intervals for the diagnosis of subacromial and subcoracoid impingement of shoulder joint. Clin. Imaging.

[11] Lo IK, Parten PM, Burkhart SS (2003). Combined subcoracoid and subacromial impingement in association with anterosuperior rotator cuff tears: An arthroscopic approach. Arthroscopy.

[12] Navarro-Ledesma S, Fernandez-Sanchez M, Struyf F, Luque-Suarez A (2022). Differences in coracohumeral distance between the symptomatic and the asymptomatic shoulder in patients with unilateral shoulder pain and in healthy participants: A cross-sectional study. J. Manipulative Physiol. Ther..

[13] Oh JH (2016). Measurement of coracohumeral distance in 3 shoulder positions using dynamic ultrasonography: Correlation with subscapularis tear. Arthroscopy.

[14] Navarro-Ledesma S, Struyf F, Labajos-Manzanares MT, Fernandez-Sanchez M, Luque-Suarez A (2017). Is coracohumeral distance associated with pain-function, and shoulder range of movement, in chronic anterior shoulder pain?. BMC Musculoskelet. Disord..

[15] Tracy MR, Trella TA, Nazarian LN, Tuohy CJ, Williams GR (2010). Sonography of the coracohumeral interval: A potential technique for diagnosing coracoid impingement. J. Ultrasound Med..

[16] Giphart JE, van der Meijden OA, Millett PJ (2012). The effects of arm elevation on the 3-dimensional acromiohumeral distance: A biplane fluoroscopy study with normative data. J. Shoulder Elbow Surg..

[17] Mozingo JD (2022). Comparison of glenohumeral joint kinematics between manual wheelchair tasks and implications on the subacromial space: A biplane fluoroscopy study. J. Electromyogr. Kinesiol..

[18] Brunkhorst JP, Giphart JE, LaPrade RF, Millett PJ (2013). Coracohumeral distances and correlation to arm rotation: An in vivo 3-dimensional biplane fluoroscopy study. Orthop. J. Sports Med..

[19] Drolet P, Martineau A, Lacroix R, Roy JS (2016). Reliability of ultrasound evaluation of the long head of the biceps tendon. J. Rehabil. Med..

[20] Navarro-Ledesma S, Struyf F, Falla D, Luque-Suarez A (2019). Non-traumatic chronic shoulder pain is not associated with changes in rotator cuff interval tendon thickness. Clin. Biomech. (Bristol, Avon).

[21] Navarro-Ledesma S (2017). Does the acromiohumeral distance matter in chronic rotator cuff related shoulder pain?. Musculoskelet. Sci. Pract..

[22] Charry FB, Martinez MJL, Rozo L, Jurgensen F, Guerrero-Henriquez J (2021). In vivo effects of two shoulder girdle motor control exercises on acromiohumeral and coracohumeral distances in healthy men. J. Man. Manip. Ther..

[23] Mackenzie TA, Bdaiwi AH, Herrington L, Cools A (2016). Inter-rater reliability of real-time ultrasound to measure acromiohumeral distance. PM R.

[24] Pijls BG, Kok FP, Penning LI, Guldemond NA, Arens HJ (2010). Reliability study of the sonographic measurement of the acromiohumeral distance in symptomatic patients. J. Clin. Ultrasound.

[25] Cavaggion C (2022). Subacromial space measured by ultrasound imaging in asymptomatic subjects and patients with subacromial shoulder pain: An inter-rater reliability study. Physiother. Theory Pract..

[26] Kottner J (2011). Guidelines for Reporting Reliability and Agreement Studies (GRRAS) were proposed. J. Clin. Epidemiol..

[27] Martinoli C (2010). Musculoskeletal ultrasound: Technical guidelines. Insights Imaging.

[28] Schmidt WA, Schmidt H, Schicke B, Gromnica-Ihle E (2004). Standard reference values for musculoskeletal ultrasonography. Ann. Rheum. Dis..

[29] Michener LA, Walsworth MK, Doukas WC, Murphy KP (2009). Reliability and diagnostic accuracy of 5 physical examination tests and combination of tests for subacromial impingement. Arch. Phys. Med. Rehabil..

[30] Razmjou H, Dwyer T, Holtby R (2018). Impact of symptom bilaterality and hand dominance on patient-reported disability outcomes. SAGE Open Med..

[31] Mintken PE, Glynn P, Cleland JA (2009). Psychometric properties of the shortened disabilities of the Arm, Shoulder, and Hand Questionnaire (QuickDASH) and Numeric Pain Rating Scale in patients with shoulder pain. J. Shoulder Elbow Surg..

[32] Roach KE, Budiman-Mak E, Songsiridej N, Lertratanakul Y (1991). Development of a shoulder pain and disability index. Arthritis Care Res..

[33] Thoomes-de Graaf M (2015). The Dutch Shoulder Pain and Disability Index (SPADI): A reliability and validation study. Qual. Life Res..

[34] McClure P, Tate AR, Kareha S, Irwin D, Zlupko E (2009). A clinical method for identifying scapular dyskinesis, part 1: Reliability. J. Athl. Train.

[35] Bujang MA, Baharum N (2017). A simplified guide to determination of sample size requirements for estimating the value of intraclass correlation coefficient: A review. Arch. Orofac. Sci..

[36] Shrout PE, Fleiss JL (1979). Intraclass correlations: Uses in assessing rater reliability. Psychol. Bull..

[37] Koo TK, Li MY (2016). A guideline of selecting and reporting intraclass correlation coefficients for reliability research. J. Chiropr. Med..

[38] Portney, L. G. *Foundations of Clinical Research: Applications to Evidence-Based Practice* (ed. Davis, F.A.) Chap. 32. 485–508. (F. A. Davis Company, 2020).

[39] Bland JM, Altman DG (1986). Statistical methods for assessing agreement between two methods of clinical measurement. Lancet.

[40] Ludbrook J (1997). Comparing methods of measurements. Clin. Exp. Pharmacol. Physiol..

[41] Connolly DJ, Berman L, McNally EG (2001). The use of beam angulation to overcome anisotropy when viewing human tendon with high frequency linear array ultrasound. Br. J. Radiol..

